# Acupuncture in diabetic peripheral neuropathy—protocol for the randomized, multicenter ACUDPN trial

**DOI:** 10.1186/s13063-021-05110-1

**Published:** 2021-02-26

**Authors:** J. Dietzel, S. Hörder, I. V. Habermann, G. Meyer-Hamme, K. Hahn, M. Ortiz, S. Roll, K. Linde, D. Irnich, M. Hammes, R. Nögel, M. Wullinger, V. Wortman, J. Hummelsberger, S. N. Willich, S. Schröder, B. Brinkhaus

**Affiliations:** 1 Institute for Social Medicine, Epidemiology and Health Economics, Charité - Universitätsmedizin Berlin, corporate member of Freie Universität Berlin, Humboldt-Universität zu Berlin, and Berlin Institute of Health, Luisenstr. 57, 10117 Berlin, Germany; 2 TCM-Zentrum am UKE, Breitenfelder Str. 15, 20251 Hamburg, Germany; 3 Department of Neurology with Experimental Neurology, Charité-Universitätsmedizin Berlin, corporate member of Freie Universität Berlin, Humboldt-Universität zu Berlin, and Berlin Institute of Health, Berlin, Germany; 4 TUM School of Medicine, Institute of General Practice, Technical University of Munich, Munich, Germany; 5 Department of Anaesthesiology, Ludwig-Maximilians University (LMU), Marchioninistraße 15, Munich, Germany; 6 Praxis Dr.med. Michael Hammes, Louisenstraße 27a, Homburg, Germany; 7 Praxis Prof. Hempen & Kollegen, Fachärztliches Zentrum, Chinesische Medizin, Akupunktur, Franz-Joseph-Straße 38, 80801 Munich, Germany; 8 Praxis für chinesische Medizin, Rathausstr. 10, 83022 Rosenheim, Germany; 9 Praxis für TCM, Dilherrstr. 6, 90429 Nürnberg, Germany; 10 International Society for Chinese Medicine, Munich, Germany

**Keywords:** Acupuncture, Chinese medicine, Peripheral neuropathy, Type II diabetes mellitus, Complementary medicine, Randomized controlled trial

## Abstract

**Background:**

Acupuncture is used to treat patients with diabetic peripheral neuropathy; however, the evidence is unclear. We present the design and methodology of the ACUDPN (ACUpuncture in Diabetic Peripheral Neuropathy) trial, which investigates the effectiveness of acupuncture for the treatment of diabetic peripheral neuropathy (DPN) symptoms. The aim of this study is to investigate whether acupuncture is effective for the treatment of DPN symptoms.

**Methods:**

This study is a two-armed, randomized, controlled, parallel group, open-label, confirmatory, multicenter trial (8-week intervention period plus 16 weeks of follow-up). Physicians in outpatient units in Germany who specialize in acupuncture treatment will treat 110 diabetes type II patients with clinical symptoms of peripheral neuropathy in the feet and legs with signs of neuropathy according to nerve conduction testing. The patients will be randomized in a 1:1 ratio to one of the following two groups: (a) semi-standardized acupuncture plus routine care or (b) routine care alone. Acupuncture will consist of 12 treatments per patient over 8 weeks. The primary outcome will be the overall DPN-related complaints in the extremities after 8 weeks as measured by the Visual Analog Scale (VAS). Further outcome measures will include DPN-related pain, the Neuropathic Pain Symptom Inventory (NPSI), Diabetic Peripheral Neuropathic Pain Impact (DPNPI) scores, and nerve conduction parameters of the sural nerve at weeks 8, 16, and 24.

**Discussion:**

The results of this trial will be available in 2021 and will help clarify whether acupuncture can be considered effective for the treatment of DPN with regard to the subdimensions of the neuropathic clinical picture.

**Trial registration:**

ClinicalTrials.gov NCT03755960. Registered on 11 August 2018.

**Supplementary Information:**

The online version contains supplementary material available at 10.1186/s13063-021-05110-1.

## Administrative information

Note: The numbers shown in curly brackets in this protocol refer to Additional file 1 item numbers. The order of the items was modified to group similar items (see http://www.equator-network.org/reporting-guidelines/spirit-2013-statement-defining-standard-protocol-items-for-clinical-trials/).

**Table:** 

Title {1}	Acupuncture in Diabetic Peripheral Neuropathy - Protocol for the Randomized, Multicenter ACUDPN Trial.
Trial registration {2a and 2b}.	ClinicalTrials.gov: NCT03755960. First registered on 11.28.2018
Protocol version {3}	Version 1.0_22102018
Funding {4}	Investigator-initiated trial. University Medicine Charité Berlin. Partially financed by Dr. Joanna Dietzel’s research grant from the Veronica and Karl Carstens Foundation.
Author details {5a}	Joanna Dietzel, MD, Institute for Social Medicine, Epidemiology and Health Economics, Charité - Universitätsmedizin Berlin, Luisenstr. 57, 10117 Berlin, Germany T.: ++49 +30 450 529 002, F.: ++49 +30 450 529 902, Email: Joanna.Dietzel@charite.de Hörder Sebastian, Institute for Social Medicine, Epidemiology and Health Economics, Charité - Universitätsmedizin Berlin, Luisenstr. 57, 10117 Berlin, Germany, Sebastian.Hoerder@charite.de Habermann Isabel, Institute for Social Medicine, Epidemiology and Health Economics, Charité - Universitätsmedizin Berlin, Luisenstr. 57, 10117 Berlin, Germany, Isabel.Habermann@charite.de Miriam Ortiz, MD, Institute for Social Medicine, Epidemiology and Health Economics, Charité - Universitätsmedizin Berlin, Luisenstr. 57, 10117 Berlin, Germany, Miriam.Ortiz@charite.de Stephanie Roll, Institute for Social Medicine, Epidemiology and Health Economics, Charité - Universitätsmedizin Berlin, Luisenstr. 57, 10117 Berlin, Germany, Stephanie.Roll@charite.de Stephan N Willich, MD, PhD, Institute for Social Medicine, Epidemiology and Health Economics, Charité - Universitätsmedizin Berlin, Luisenstr. 57, 10117 Berlin, Germany, Stefan.Willich@charite.de Benno Brinkhaus, MD, PhD, Institute for Social Medicine, Epidemiology and Health Economics, Charité - Universitätsmedizin Berlin, Luisenstr. 57, 10117 Berlin, Germany, Benno.Brinkhaus@charite.de Gesa Meyer-Hamme, MD, TCM-Zentrum am UKE, Breitenfelder Str. 15, 20251 Hamburg, Germany, meyer-hamme@tcm-am-uke.de Sven Schröder, MD, PhD, TCM-Zentrum am UKE, Breitenfelder Str. 15, 20251 Hamburg, Germany, Lehre@tcm-am-uke.de Katrin Hahn, MD, PhD, Department for Neurology, Charité - Universitätsmedizin Berlin, Germany, Katrin.Hahn@charite.de Klaus Linde, MD, PhD, Technical University of Munich, TUM School of Medicine, Institute of General Practice, Munich, Germany, Klaus.Linde@mri.tum.de Dominik Irnich, MD, PhD, Department of Anaesthesiology, Ludwig-Maximilians University (LMU), Marchioninistraße 15, 81377 Munich, Germany, dominik.irnich@med.uni-muenchen.de Michael Hammes, MD, Praxis Dr.med. Michael Hammes, Louisenstraße 27a, 61348 Bad Homburg, Germany praxis@hammes-akupunktur-neurologie.de Rainer Nögel, MD, Praxis Prof. Hempen & Kollegen, Fachärztliches Zentrum – Chinesische Medizin, Akupunktur, Franz-Joseph-Straße 38, 80801 Munich, Germany, noegel@tcm.edu Michael Wullinger, MD, Praxis für chinesische Medizin, Rathausstr. 10, 83022 Rosenheim, Germany, info@wullinger.de Velia Wortman, MD, Praxis für TCM, Dilherrstr. 6, 90429 Nürnberg, Germany, velia@t-online.de Jospeh Hummelsberger, MD, International Society for Chinese Medicine, Franz-Joseph-Straße 38, 80801 Munich, Germany, josef@hummelsberger.net
Name and contact information of the trial sponsor {5b}	Investigator-initiated trial. Principal Investigator: Benno Brinkhaus, MD, PhD, Institute for Social Medicine, Epidemiology and Health Economics, Charité - Universitätsmedizin Berlin, Luisenstr. 57, 10117 Berlin, Germany, Stefan.Willich@charite.de Research grant by Karl und Veronica Carstens-Stiftung, Am Deimelsberg 36, 45276 Essen, Germany
Role of sponsor {5c}	Investigator-initiated trial. The principal investigator has ultimate authority over the collection, management, analysis, and interpretation of the data; writing of the report; and the decision to submit the report for publication.

## Background and rationale {6a}

Approximately 14–30% of all diabetic patients in Germany are affected by sensorimotor neuropathy [1]. Furthermore, neuropathy has been related to impaired glucose tolerance and can appear before the onset of diabetes [2].

In addition to chronic discomfort or pain, the destruction of nerve fibers can lead to the complete loss of sensation in the feet and legs, which increases the risk of diabetic foot syndrome [3]. Minor plantar cuts and trauma are overlooked and remain untreated. Due to a concomitant impairment in vascularization, wounds do not heal well, and surgical interventions as invasive as repeated amputations can become necessary [4]. Diabetic foot syndrome has dermatological, atherosclerotic, and orthopedic dimensions, and in 85–90% of cases, sensorimotor peripheral and distal symmetric neuropathy is present [5].

The authors of a Cochrane meta-analysis concluded that even strict management of blood sugar levels could not reverse or stop the destruction of nerve fibers in type II diabetes [6]. Thus far, only symptomatic treatment has been implemented for DPN [7]. The pharmacological approach for paresthesia or the painful symptoms of DPN consists of analgesics, including anticonvulsants and antidepressants, such as amitriptyline, duloxetine, pregabalin, gabapentin, and opioids. The reduction in DPN symptoms with the help of these pharmaceuticals has been reported to be insufficient due to the lack of alleviation of numbness, and all drug options have a relevant risk of side effects and drug interactions [5], which is important for patients with metabolic syndromes who experience polypharmacy as it is also necessity to treat hypertension, hyperlipidemia, and hyperglycemia. Due to the lack of therapeutic solutions for neuropathy, an increasing number of patients are considering complementary medicine, i.e., acupuncture [8].

Acupuncture is a relatively safe treatment option that has been proven to be effective for chronic pain syndromes [9]. Chinese medicine (CM) textbooks classify neuropathies under the symptom “numbness of the four extremities”, which they explain mainly with (a) qi and blood deficiency, (b) damp heat or phlegm blocking the channels, or (c) cold that has entered the meridians. They not only contain advice regarding the symptomatic treatment of neuropathy with acupuncture but also offer a more complex solution for treating diabetic nerve destruction with a combination of dietetic and herbal prescriptions, exercise, and acupuncture [10].

Scientific evidence supporting the usefulness of such treatments in DPN remains lacking, leading to the need for well-designed clinical trials.

A systematic review involving a meta-analysis of 14 randomized controlled trials (RCTs) and 1 long-term follow-up study investigating the use of acupuncture for miscellaneous neuropathies suggests that acupuncture is effective for diabetic neuropathy, Bell’s palsy, and carpal tunnel syndrome (CTS) [11]. In 4 trials investigating diabetic neuropathy and 4 trials investigating CTS, nerve conduction studies were performed; a series of acupuncture treatments produced significantly improved effects on motor nerve function parameters in the median, ulnar, and peroneal nerves. A sensory nerve conduction study (NCS) revealed that acupuncture increased the nerve conduction velocity (NCV) in the median and peroneal nerves. In a recently published RCT, 172 patients with diabetic neuropathy received 10 acupuncture treatments or 10 laser acupuncture or 10 placebo laser treatments; sural SNAP and sural and tibial NCV improved significantly when comparing needle acupuncture to placebo [12]. Neuropathies with different etiology have been investigated in the past. In a study with a design similar to our design with a randomized waiting-list control group for effectiveness, 87 Chinese patients with chemotherapy-induced neuropathy were investigated. Within this cohort, after 8 weeks, the 10 acupuncture treatments resulted in significant clinical improvements in the primary outcome (pain), clinical neurological assessment, quality of life domains, and symptom distress [13]. A tested subset of patients had no or only borderline electrophysiological signs of neuropathy at baseline. Their parameters did not change after 8 weeks. Another small pilot study investigating chemotherapy-induced neuropathy described improved NCV parameters in 11 patients who agreed to undergo acupuncture, and the parameters were compared to those of 11 control patients who received the best medical care alone [14]. To determine whether neurophysiological changes reflect neurological rehabilitation, further well-designed controlled trials using neuropathy-specific, validated questionnaires in combination with electrophysiological data as outcome parameters are needed [15].

### Objectives {7}

The aim of this randomized controlled clinical study is to assess the effectiveness and safety of acupuncture in addition to routine care compared to routine care alone for the treatment of symptomatic DPN with respect to the postulated underlying TCM-patterns in terms of the impact on overall symptoms, pain, neurophysiological outcomes, the use of analgesics, sleep disturbances, specific and general quality of life, and adverse events.

### Trial design {8}

The ACUDPN study is a two-armed, randomized, controlled, parallel group, open-label, confirmatory, multicenter clinical trial investigating the effectiveness of acupuncture plus routine care vs. routine care alone in patients with DPN.

## Methods: participants, interventions, and outcomes

### Study setting {9}

**Fig. 1:**
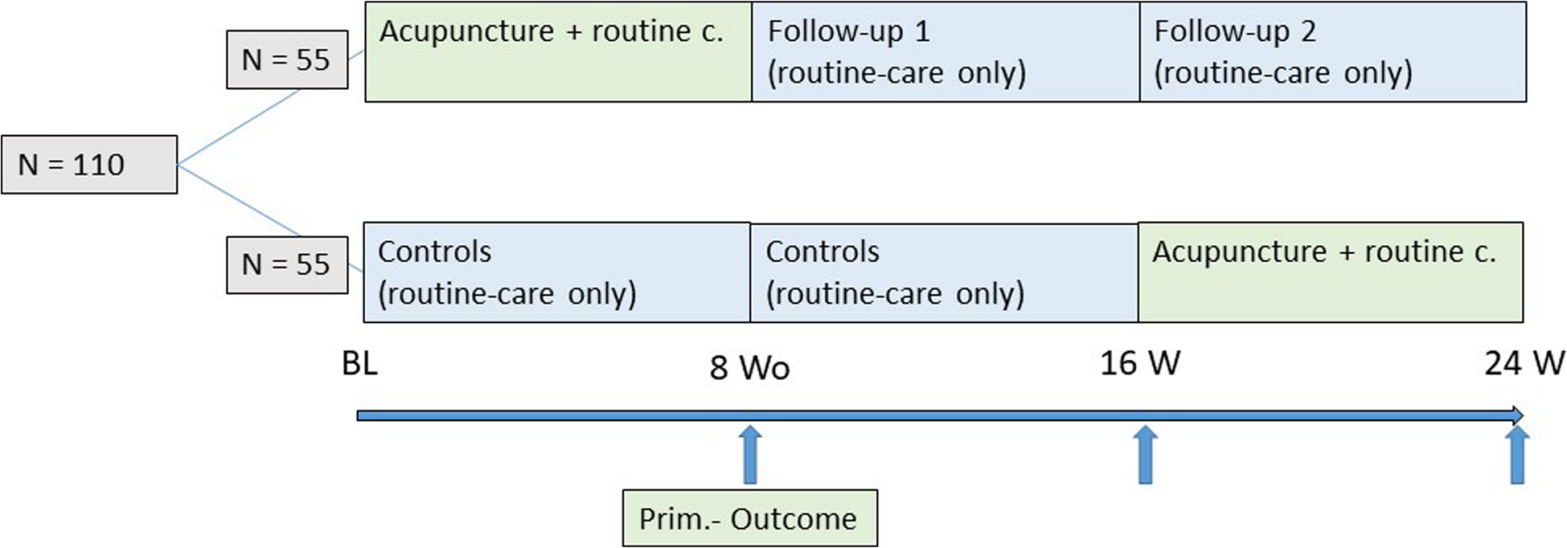
Participant timeline {13}. Time schedule, design, and outcome parameters of the ACUDPN study. *BL*, baseline; *W8*, *W16*, etc., week 8, week 16, etc., after inclusion in the study. Outcome measures: VAS pain and VAS overall DPN-related complaints, German versions of the Neuropathic Pain Symptom Inventory (NPSI), Diabetic Peripheral Neuropathic Pain Impact (DPNPI) measure, the emotional pain perception scale (SES), the SF-12 QoL, the Patient Global Impression of Change (PGIC), and neurological exam with clinical Total Neuropathy Score (cTNS)

This study is being conducted in Germany. The main recruiting center is located in Berlin at the Charité University Hospital, and another recruiting center is located in Hamburg in a research and treatment center for traditional Chinese medicine. The overall study duration per patient is 24 weeks. After randomization at baseline, the patients in the acupuncture group will undergo 12 treatment sessions in addition to routine care during the first 8 weeks of the study, followed by 16 weeks of follow-up. In the control group, all patients will continue to receive routine care and undergo several study follow-up examinations during the first 16 weeks. In this control group, patients are on a waiting list for the acupuncture treatment, which will be started at week 16 and continued until the final follow-up at week 24 using the same acupuncture treatment protocol being performed in the other study arm (see Fig. 1 and Table 1).

**Table 1:**
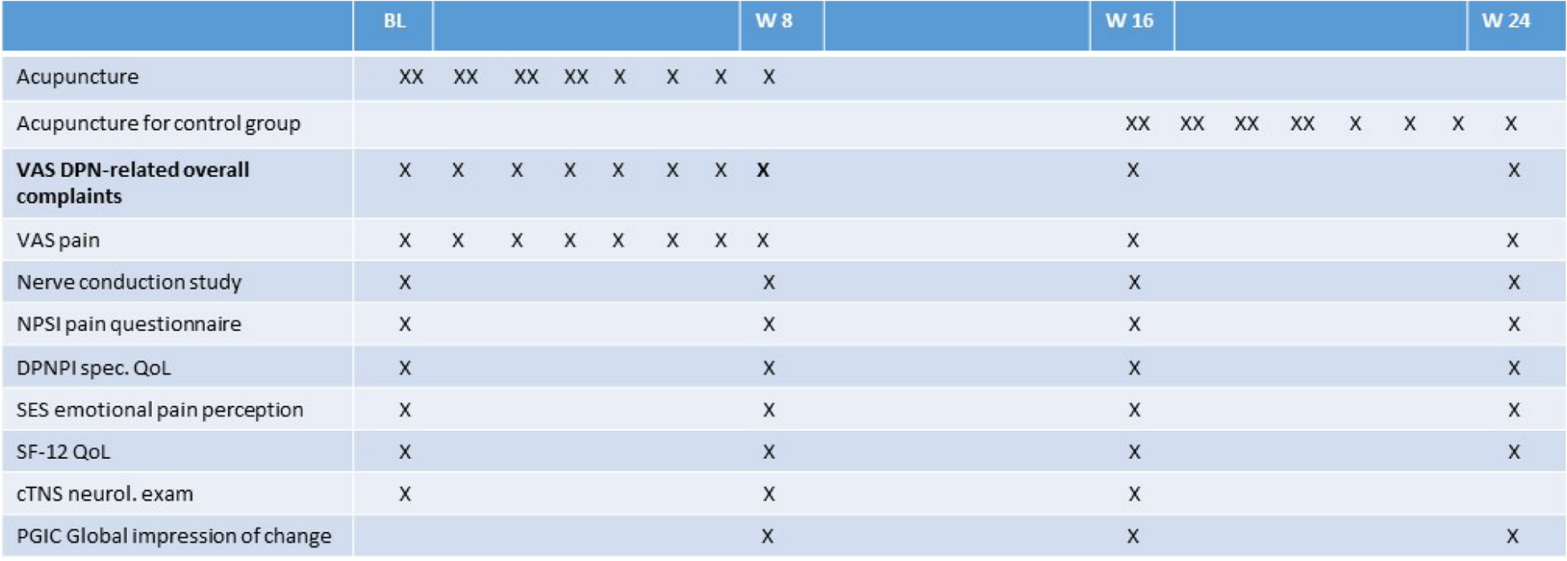
Participant timeline {13}. Time schedule, design, and outcome parameters of the ACUDPN study. *BL*, baseline; *W8*, *W16*, etc., week 8, week 16, etc. after inclusion in the study. Outcome measures: VAS pain and VAS overall DPN-related complaints, German versions of the Neuropathic Pain Symptom Inventory (NPSI), Diabetic Peripheral Neuropathic Pain Impact (DPNPI) measure, the emotional pain perception scale (SES), the SF-12 QoL, the Patient Global Impression of Change (PGIC), and neurological exam with clinical Total Neuropathy Score (cTNS)

### Patients and recruitment {15}

Patients’ enrollment began in February 2019. The participants are recruited by advertisements in public transportation units and the distribution of flyers in doctors’ and podiatrists’ offices and point-of-care facilities for patients with diabetes in Berlin and Hamburg. An incentive of 50€ has been offered to physicians or podiatrist for each patient referred by their practice who could be successfully recruited for the trial.

### Eligibility criteria {10}

To participate in the study, patients must meet the following inclusion criteria: female or male patients (aged 18–80 years) with symptoms of at least moderate diabetic peripheral neuropathy, patients with a diagnosis of diabetic peripheral neuropathy due to diabetes mellitus type II, patients with minimum overall DPN-related complaints of at least 40 mm on a 0–100 mm VAS, patients with a pathological NCV < 42 m/s and/or an amplitude of the sural nerve< 6 μV, and patients who have completed titration of pain medication against DPN. In addition, patients must be able to complete a diary for the self-evaluation of symptoms, must record the use of symptomatic medication, and must provide written informed consent.

The main exclusion criteria for the patients are as follows: severe DPN with muscular weakness of the proximal leg muscles; neuropathy due to other reasons (such as borrelia infection, human immunodeficiency virus infection, hereditary factors, alcohol, or a history of neurotoxic drug use); anticoagulation or bleeding disorders; severe peripheral artery disease in Fontaine stage IV; ulcers or gangrenous lesions of the feet; severe fatigue syndrome; traumatic lesions of the nerves or vessels in the lower extremities; opioid use before inclusion in the study; regular use of cannabis or cannabinoids; lipoic acid infusions planned during participation in the trial; scheduled psychotherapy during study participation; additional therapy with complementary medicine or physical therapy for symptoms of DPN during the 6 weeks before inclusion in the study or planned during the study; obesity (BMI > 35 kg/m^2^); and pregnancy or lactation. No restrictions will be made regarding the underlying disease pattern according to the TCM diagnosis, though patterns will be registered.

### Informed consent {26a}

Written and oral informed consent will be obtained by the study’s physicians.

### Additional consent provisions for the collection and use of the participants’ data and biological specimens {26b}

On the consent form, the participants will be asked if they agree to the use of their data should they choose to withdraw from the trial. The participants will also be asked for permission allowing the research team to share relevant data with people from the universities participating in the research or regulatory authorities when relevant. This trial does not involve collecting biological specimens for storage.

### Randomization

#### Sequence generation {16a}

Individual patient randomization at a 1:1 ratio into the two study arms will be performed via a computer-generated randomization list (prepared by SAS 9.4, SAS Institute Inc., Cary, NC, USA), which will be kept at the study center in Berlin. Randomization will be stratified by the study center.

This computer-generated list shows only one result at a time and is unavailable to the enrolling study physician.

#### Concealment mechanism {16b}

Randomization is performed by the coordinating study nurse using the computer-generated randomized list (prepared by SAS 9.4, SAS Institute Inc., Cary, NC, USA); the computer reveals only one result at a time. The results of the randomization are provided via telephone to the study physician once he declares that the patient is eligible for participation.

#### Implementation {16c}

If the study physicians declare a patient eligible for enrollment, they will contact the coordinating study nurse. The nurse will generate the allocation and communicate the result to the physician via telephone and fax. Then, the physician will communicate the result of the randomization to the patient.

### Assignment of interventions: blinding {17a}

This study is an open-label study, and the participants and physicians will be aware of the treatment allocation. Only the data analysts and statisticians are blinded.

### Procedure for unblinding if needed {17b}

The design is open label, and only the statisticians will be blinded until the end of the analysis; thus, unblinding will not occur.

### Study intervention {11a}

**Fig. 2:**
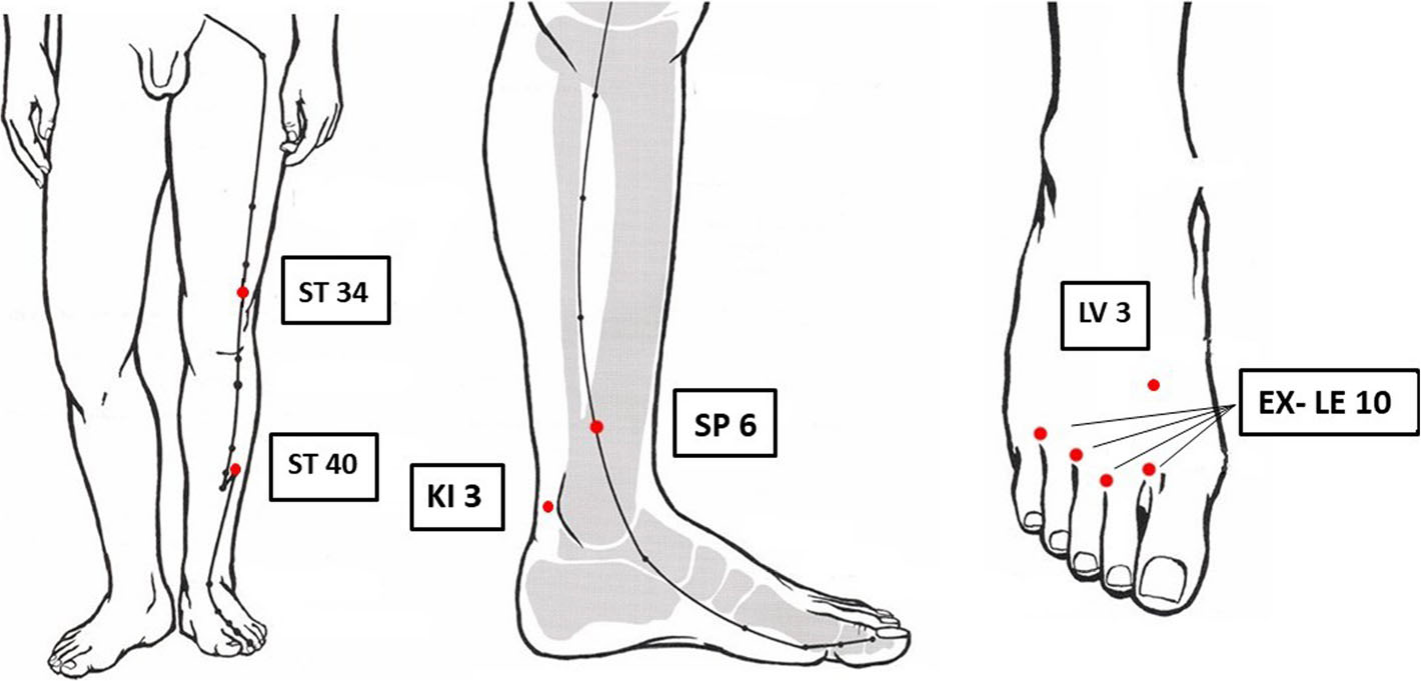
Obligatory acupuncture points used in the ACUDPN study (modified from Stux, G. Akupunktur Lehrbuch und Atlas, Springer Verlag Berlin Heidelberg, 2008 with permission)

Two neurologists and six examined acupuncturists (four licensed physicians with experience in TCM- research and two doctoral students with diplomas for acupuncture and 140 h of acupuncture training) are presently recruiting and treating patients. The choice of acupuncture points was made via discussion with six acupuncture experts from the following two major German societies for medical acupuncture: the German Medical Acupuncture Association (*Deutsche Ärztegesellschaft für Akupunktur*; DÄGfA), Munich, and the International Society for Chinese Medicine (*Societas Medicinae Sinensis,* SMS), Munich. The ultimate strategies were generally considered a pragmatic compromise between the need for standardization in science and the need for individualization in Chinese medicine. Furthermore, the rationale of the acupuncture- point selection was furthermore supported by the treatment strategy provided in a pilot study from Meyer-Hamme et al. [16]. The acupuncture treatment consists of 12 sessions administered over a period of 8 weeks (preferably 2 sessions in each of the first 4 weeks, followed by 1 session per week in the remaining 4 weeks). The acupuncture treatment in the ACUDPN study is semi-standardized (Table 2) as follows: all patients must be treated at the basic obligatory acupuncture points, i.e., bilaterally ST34, ST40, SP6, Ki 3, and LV3, and bilaterally at the EX-LE-10 Bafeng points, which sum up to 18 needles per session (see Fig. 2).

**Table 2:** Acupuncture points used in the ACUDPN study

**Obligatory points**	ST 34, ST 40, SP 6, KI 3, LV 3, EX-LE-10 Bafeng
**Optional points depending on the following Chinese medicine diagnoses**	
Cold invasion into meridians	Moxa on EX-LE-10 Bafeng, KI 3
Deficiency of qi and xue	ST 36, GB 39, ST 41 (in pronounced sensory ataxia)
Qi-stagnation and blood stasis	GB 34, GB 40, SP 4
Wind and phlegm block channels	ST 36, SP 9, SP 4
Phlegm and heat block channels	SP 9, SP 10, GB 40, KI 7

Furthermore, at least 2 of 10 facultative acupuncture points will be selected according to the patient’s individual constitution and disease pattern following the diagnostic principles of Chinese medicine {11b}. The acupuncture points used in a session will be documented each time. A diagnosis of traditional Chinese syndrome at baseline is mandatory, and the diagnosis will be documented. Patients will lie down in supine position. Needles will be inserted perpendicularly and stimulated only in the beginning to achieve a DeQi sensation and then will be left in place without further stimulation for 20–25 min.

The duration of 12 acupuncture sessions within one treatment cycle with 1–2 sessions per week and between 20 and 30 min per session is supported by common textbooks for acupuncture and taught in acupuncture schools internationally. The needle length and diameter will be chosen according to the anatomical location and constitutional picture. If numbness is present, superficial puncture with a sharp needle will be carried out at the Bafeng points, and tiny amounts of blood will be released twice during the treatment period. In both groups, moxibustion is allowed and will be applied by using a common moxibustion TDP- lamp (Phoenix Bio TDP CE005) exclusively at the feet over 15 min at 20 cm distance to the toes, in case of cold and numb feet. Since the focus is on body acupuncture, we excluded auricular acupuncture strategies.

The patients in the control group will not receive acupuncture treatment for a period of 16 weeks after randomization. During this period, the patients in the control group will participate in follow-ups only with the use of diaries and questionnaires. After 16 weeks, the patients in this group will receive the same acupuncture treatment according to the principles described above.

The patients in both groups will be allowed to continue routine care medication (if applicable), which will be adapted as needed during the study.

#### Explanation of the choice of comparators {6b}

To determine whether acupuncture has overall effects on DPN, we compare acupuncture plus routine care versus routine care in this confirmatory trial. If we yield positive results, acupuncture should be compared in another clinical trial with sham acupuncture to study the specific effect of acupuncture.

#### Criteria for discontinuing or modifying the allocated interventions {11b}

If a patient wishes to drop out, serious side effects occur, or any circumstances place the patients’ health at hazard, the physician will discontinue the treatment. The allocated interventions will not be modified.

#### Strategies to improve adherence to interventions {11c}

Adherence to treatment requires the physical presence of the patient. The phone numbers of the patients have been registered. If a patient does not show for an appointment or does not return the questionnaires or the questionnaires are incomplete, the study nurse will contact the participant. If necessary, a treating physician will speak with the participant.

#### Relevant concomitant care permitted or prohibited during the trial {11d}

Permitted concomitant care comprises all drug prescriptions for DPN pain, except for drugs belonging to complementary medicine (i.e., traditional Chinese herbs). The prohibited care includes the uptake of new physiotherapeutic or psychotherapeutic measures aiming to ease DPN-related discomfort.

#### Provisions for post-trial care {30}

Both trial centers’ insurance will compensate for those who suffer harm from trial participation.

### Outcomes {12}

The primary outcome parameter in the ACUDPN study is the overall DPN-related complaints at week 8 measured on a 0–100 mm VAS (0 = no complaints, 100 = worst imaginable complaints).

The VAS is a well-established and validated assessment tool that has been used in several clinical studies in conventional medicine and complementary medicine, e.g., acupuncture [11, 17], as a primary or secondary outcome measure.

Responders to the study intervention will be defined as patients with an overall change in DPN-related complaints of 15 mm or more between the VAS at baseline and the VAS in week 8.

The secondary outcome measures include the overall number of DPN-related complaints at week 16 and week 24 (VAS); the pain intensity at weeks 8, 16, and 24 (VAS); and the Neuropathic Pain Symptom Inventory (NPSI) score at weeks 8, 16, and 24. The NPSI scores subdimensions of neuropathic pain, such as spontaneous ongoing or paroxysmal pain, evoked pain (i.e., mechanical and thermal allodynia/hyperalgesia), and dysesthesia/paresthesia, using an 11-point scale [18].

Since numbness is not assessed in the NPSI, we added the 11-point NRS scale for numbness (0 = no numbness at all to 10 = no sensation on the soles of the feet).

The Diabetic Peripheral Neuropathic Pain Impact (DPNPI) measure is a validated tool used to assess the disease-specific impact on the quality of life [19] and will be applied at the abovementioned time points in addition to a general health-related quality of life assessment using the SF-12 [20].

The German questionnaire on emotional pain perception *Schmerz Empfindungs Skala* (SES) will be used to monitor the effect of acupuncture on the affective components of pain [21] following a timely schedule as described above, i.e., at baseline and weeks 8, 16, and 24.

The Patient Global Impression of Change (PGIC) scale consists of a patient assessment from 0 = much worse to 7 = much better [22] and will be measured only at weeks 8, 16, and 24.

Further secondary outcome parameters are the general clinical and neurological presentation registered with the Neuropathy Deficit Score (NDS) [23] and the clinical Total Neuropathy Score (cTNS) [24]; these assessments will be noted as described above at baseline and weeks 8, 16, and 24. Adverse events, dropouts, withdrawals, and the respective reasons will be documented.

In addition, the patients will be requested to complete patient diaries following a daily schedule, which will include recording on-demand medication use, a weekly assessment of the average VAS score of pain, and the overall number of DPN-related complaints during the first 8 weeks and at weeks 15 to 16 and 23 to 24.

The neurophysiological parameters will include measurements of the sural nerve. This nerve is pathognomonically affected in diabetic neuropathy and is used to monitor peripheral neuropathy. The nerve conduction velocity and amplitude of sensible action potentials will be measured with a novel point-of-care nerve conduction device (POCD) produced by NeuroMetrix™, Texas. This device is used in the DPNCheck®, which provides accurate, cost-effective, and quick screening data for the diagnosis and monitoring of diabetic peripheral neuropathy (DPN). With a 10-s period of measurement, this device can provide the numeric values of velocity (m/s) and amplitude (μV). This device has been validated in several studies as an accurate screening and monitoring instrument for DPN and the evaluation of the risk of diabetic foot [25–27].

TCM-related indexes as outcomes are not used in this trial.

### Data collection and management

#### Plans for the assessment and collection of outcomes {18a}

The baseline and follow-up neurological examinations, including the electrophysiological data collection, will be performed by two neurologists. These neurologists do not perform any acupuncture treatments. Each acupuncture treatment is documented by trained acupuncturists; the needles at mandatory and facultative points and their mode of application (i.e., stimulating or sedating technique) are noted and counted.

Medication will be listed on a medication log; any changes in pain-modifying drugs will be noted on a weekly basis in the diaries. At the baseline visit, the patients are given diaries that they take home for the first 8 weeks, and weekly outcomes are to be noted in these diaries. Every 8 weeks, via mail, the patients receive another set of questionnaires to assess the abovementioned outcomes. The diaries and questionnaires must be returned in the envelopes provided by the study center.

To prevent loss by mail, data sheets of the baseline examination are provided in duplicates; one copy remains at the study site. Upon receipt of the baseline and follow-up case report forms and the patients’ diaries and questionnaires at the main study center at the Charité University Hospital, the monitoring study staff checks for the completeness of the data. The responsible physician will be notified of any missing data from the physical or neurological evaluation within the next working day. All questionnaires and dairies are transmitted via mail; the delayed return of the questionnaires will be followed by a reminder phone call to the patient. Missing outcomes in the questionnaires will be completed by contacting the patients and sending the patients a copy of the incomplete questionnaire or isolated questions via mail.

#### Plans to promote participant retention and complete follow-up {18b}

Protocol deviations are documented on a separate list. The patients have been informed of the value of completing the study course. The patients receive reminding phone calls if they miss an acupuncture treatment or a questionnaire has not been returned in time. If a patient drops out of treatment, it will be documented on a special study discontinuation form, and the patient will be motivated to continue to complete the questionnaires. After receipt of the final questionnaire, the patients will be reimbursed for transportation fees to the study site for the 12 acupuncture sessions and 2 follow-up examinations with the lump sum of 60€.

#### Data management {19}

After checking for completeness and quality, the data will be entered in electronic databases by designated staff at the main study center at the Charité University Hospital. In a separate step, the correct transfer (e.g., double entries and range checks of the data values) and coding of the data will be checked by a different set of people. All data are archived in a standardized way and will be deleted after 10 years. The reference procedures are provided in the protocol and are consistent with the standard operation procedures of the investigating institute of the Charité University Hospital.

#### Confidentiality {27}

The study data are documented and archived using pseudonyms. During the study, all personal information will be stored in a locked, password-accessible MS-ASSESS database. Paper forms containing personal information are locked in a metal cabinet and will be destroyed along with the deletion of electronic information after the completion or discontinuation of the study. The concept of data safety had to be presented to the university responsible for data safety and follows the standards of the German legal guidelines for data safety and the Federal State of Berlin.

#### Plans for the collection, laboratory evaluation, and storage of biological specimens for genetic or molecular analysis in this trial/future use {33}

No biological specimens will be collected.

### Statistical analysis

#### Sample size {14}

A sample size of 90 patients (45 per group) will provide 80% power to detect a difference in the mean values between the acupuncture group and the control group of at least 15 mm on the VAS (DPN-related overall complaints) after 8 weeks with an anticipated common standard deviation of 25 mm using a 2-sided *t* test with a level of significance of alpha = 0.05. To account for a dropout rate of approximately 15%, 110 patients must be randomized.

#### Statistical methods for the primary and secondary outcomes {20a}

The changes in the overall number of DPN-related complaints (measured by the VAS) between baseline and week 8 will be used as the primary outcome parameter. The primary analysis of the primary endpoint will be conducted with an analysis of the covariance (ANCOVA). The treatment group (acupuncture/control) and study center will be included as fixed-effect factors in the model, and the baseline value of the overall number of VAS-DPN-related complaints will be used as a fixed covariate. The adjusted means will be derived from this model and presented along with the two-sided 95% confidence intervals in each treatment group and the *p* value of the group comparison (significance level of 5%, two-sided). The calculation will be performed using the full analysis set (FAS) based on the intention-to-treat-principle (ITT), thus evaluating each patient in the treatment group at random without the replacement of missing values. All further analyses will be considered exploratory (without adjustment for multiple testing).

The secondary outcomes will be analyzed similarly with an ANCOVA or logistic regression (depending on the scale of the outcome) and will include the treatment group and study center as fixed-effect factors and the respective baseline value (where applicable) as a fixed covariate.

#### Interim analysis {21b}

An interim analysis is not planned. If the study has to be discontinued, the interim results will be accessible only to the principal investigators, who will make a decision regarding publication.

#### Methods for additional analyses {20b}

A subgroup analysis is planned to determine the effects of acupuncture on the subdimensions of neuropathic dysesthesias, such as tingling, numbness, and pain.

#### Methods applied in the analysis to address protocol nonadherence and any statistical methods applied to address missing data {20c}

The analysis will be performed with the full analysis set (FAS) based on an intention to treat (ITT). Missing values will not be replaced. Further analyses will be regarded as explorative (without adjustment and multiple imputation).

#### Plans to provide access to the full protocol, participant-level data, and statistical code {31c}

The detailed information of the study is accessible in the study registry clinicaltrials.gov. After publication, full access to the participant level data and statistical code is planned by the Research Institute of Social Medicine, Epidemiology and Health Economics of the Charité University Hospital Berlin.

### Oversight and monitoring

#### Composition of the coordinating center and trial steering committee {5d}

The coordinating center is located at the research institute (Institute for Social Medicine, Epidemiology and Health Economics) at the Charité University Hospital in Berlin and comprises the principal investigator, the coordinating physician, 4 acupuncturists, 2 study nurses, the data monitoring and management team, and the statistical advisor. The steering committee consists of the director of the research institute and the principal investigator. Serious adverse events are directly reported to this committee within 24 h of notification.

#### Composition of the data monitoring committee, its role, and reporting structure {21a}

A data monitoring committee was not considered as this study adopts a low-risk intervention, and an interim analysis is not planned in this study.

#### Adverse event reporting and harm {22}

A short description of the patients’ general wellbeing and any adverse events is documented at each of the 12 acupuncture sessions. Serious adverse events are noted on a separate form and will be reported to the principal investigator within 24 h. Known potential side effects are intensified tingling at the beginning of treatment (in patients with underlying tingling sensation); local pain, local infection, or bruising at the needle site; and orthostatic reaction after rising from the reclined position.

#### Frequency and plans for auditing trial conduct {23}

The project management group meets every week to discuss the trial development and upcoming issues in both participating centers. The study documents and facilities were audited in October 2019; the auditor is independent from the research institute of the Charité University Medicine Hospital; no major findings were documented. All study procedures have been described in SOPs to maintain the quality of the trial conduct.

#### Plans for communicating important protocol amendments to relevant parties (e.g., trial participants and ethical committees) {25}

All amendments to the protocol are communicated via weekly research team meetings; the center in Hamburg is informed via E-mail, telephone conference, and a personal visit regarding any changes to the protocol. All amendments must be presented to the local ethical board in Hamburg for a positive vote as required by the federal organization of the ethical boards in Germany. If further changes are made, they will be communicated in the same way.

#### Dissemination plans {31a}

The participants are allowed to ask for the results of their individual electrophysiological examinations of the nervus suralis as soon as they completed their final study procedures. The trial participants will be informed of the general outcome of the study via email as soon as the results have been published in a peer reviewed journal. The Veronica and Karl Carstens foundation, who provided a research grant to Dr. Joanna Dietzel, will publish the results and a link to the publication on their website.

## Discussion

This study involving type II diabetic patients who suffer from diabetic neuropathy investigates whether 12 sessions of acupuncture along with routine care can improve symptoms in the lower limbs compared to the use of routine care alone. To the best of our knowledge, the ACUDPN study is the first clinical study to investigate the effectiveness of acupuncture treatment for DPN compared to routine care alone in a multicenter two-armed clinical trial. Due to the variety of DPN-related neurological complaints, the effectiveness of acupuncture is not easily investigated in this condition. We decided to test the overall VAS-DPN-related complaints to account for not only pain but also tingling and numbness as these symptoms can also seriously affect DPN patients. However, to allow for the differentiation of the subdimensions of neuropathic pain, the NPSI will be used in this study as a secondary outcome parameter.

The neurophysiological follow-up is limited to measurements of the sural nerve with the new handheld device DPNCheck®. The conductivity of the sural nerve is an established indicator of the severity of DPN and can be assessed noninvasively. The advantage of the new device is the provision of numeric values, leaving no room for the interpretation of curves as is usually performed in conventional manual evaluations of the measurement.

The main inclusion criteria have been applied in other clinical studies before evaluating the efficacy of acupuncture on the basis of nerve conduction measurements [11, 13, 16]. Therefore, in addition to using a modified and extended inventory of tools, our study fulfills the confirmatory demands suggested by previous research.

Regarding the weak evidence of an acupuncture effect on DPN in prior sham-controlled trials [28], most trials suffered from methodological shortcomings; therefore, a repetition of the question in an open-label trial of higher methodological quality comparing routine care with acupuncture to routine care alone seems well justified. Therefore, we decided to clarify primarily whether acupuncture has overall effects on DPN in a routine care study. As a second step, a sham-controlled trial should be designed to determine whether acupuncture has specific needling-related effects on DPN.

Acupuncture practiced according to the principles of Chinese medicine is an individualized therapy [29]. In the ACUDPN study, we use a semi-standardized treatment protocol that is similar to the treatments applied in several other previous studies, including the ART and the ACUSAR studies [30–34].

Compared to previous studies investigating acupuncture for the treatment of DPN, the ACUDPN study uses additional acupuncture sessions and applies a more rigorous methodology, including validated questionnaires and neurophysiological parameters, with a longer follow-up period to assess the long-term effects.

In conclusion, the results of the ACUDPN study will have an impact on the decision of whether acupuncture might be considered an effective and safe therapeutic option for the treatment of DPN and whether acupuncture can exert neuroregenerating effects.

## Trial status

Protocol Version 1.0_22102018. Recruitment started in February 2019. Number of included patients on Jan 5, 2021: 62. Planned end of the recruitment period: Oct 2021 (due to the delay caused by the coronavirus pandemic) with inclusion of the last patient; planned end of study: March 2022.

## Supplementary Information

**Additional file 1.** SPIRIT 2013 Checklist: Recommended items to address in a clinical trial protocol and related documents*.

## Abbreviations

ACUDPN: Acupuncture in Diabetic Peripheral Neuropathy
ACUSAR: Acupuncture in Seasonal Allergic Rhinitis
ART: Acupuncture randomized trial
DPN: Diabetic Peripheral Neuropathy
DPNPI: Diabetic Peripheral Neuropathic Pain Impact
NCV: Nerve conduction velocity
NCS: Nerve conduction study
NDS: Neuropathy Deficit Score
NPSI: Neuropathic Pain Symptom Inventory
NSS: Neuropathy Symptom Score
PGIC: Patient Global Impression of Change
SES: Schmerzempfindungs Skala -German Emotional Pain Perception Scale
SF-12 QoL: Short-Form-12 Quality of Life Questionnaire
cTNS: Clinical Total Neuropathy Score
VAS: Visual Analog Scale
